# Dr. Matthew R. Carson

**Published:** 1914-11

**Authors:** 


Dr. Matthew R. Carson, Albany, 1857, died at his home in Canandaigua, October 6, aged 78. He was born in the town of Seneca, Ontario Co., May 25, 1836, the son of Robert Carson, Jr. He graduated from the Canandaigua Academy. After practicing in Albany for a year, he returned to Canandaigua and remained in practice till a few years ago. He was a charter member of the Canandaigua Society of Physicians and Surgeons, which he entertained recently, in his home, on the occasion of its semi-centennial anniversary. He had also been President of the Ontario County Medical Society, a token of the esteem of his associates, which was shared by all. Beside other children, he leaves a son in the medical profession, Robert of Rochester and a brother James C., until recently Superintendent of the Syracuse State Hospital.

